# Smart Skin Patterns Protect Springtails

**DOI:** 10.1371/journal.pone.0025105

**Published:** 2011-09-30

**Authors:** Ralf Helbig, Julia Nickerl, Christoph Neinhuis, Carsten Werner

**Affiliations:** 1 Max Bergmann Centre of Biomaterials, Leibniz Institute of Polymer Research Dresden, Dresden, Germany; 2 Institute of Botany, Technische Universität Dresden, Dresden, Germany; 3 B CUBE Innovation Centre for Molecular Bioengineering, Technische Universität Dresden, Dresden, Germany; Massey University, New Zealand

## Abstract

Springtails, arthropods who live in soil, in decaying material, and on plants, have adapted to demanding conditions by evolving extremely effective and robust anti-adhesive skin patterns. However, details of these unique properties and their structural basis are still unknown. Here we demonstrate that collembolan skin can resist wetting by many organic liquids and at elevated pressures. We show that the combination of bristles and a comb-like hexagonal or rhombic mesh of interconnected nanoscopic granules distinguish the skin of springtails from anti-adhesive plant surfaces. Furthermore, the negative overhang in the profile of the ridges and granules were revealed to be a highly effective, but as yet neglected, design principle of collembolan skin. We suggest an explanation for the non-wetting characteristics of surfaces consisting of such profiles irrespective of the chemical composition. Many valuable opportunities arise from the translation of the described comb-like patterns and overhanging profiles of collembolan skin into man-made surfaces that combine stability against wear and friction with superior non-wetting and anti-adhesive characteristics.

## Introduction

**Figure 1:**
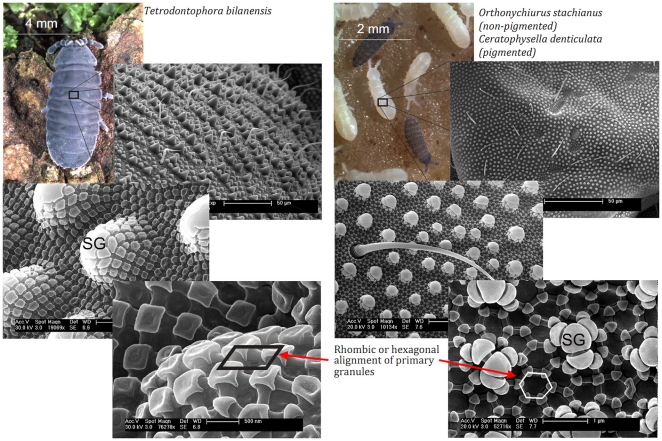
Springtail skin combines bristles and a unique nanoscopic comb pattern. The rhombic or hexagonal comb pattern is formed by small primary granules connected by ridges. Additionally, some - but not all - species possess papillous secondary granules (SG), which can significantly differ in shape, depending on the specific habitat and body size of the respective species [for details see **Information S1**].

Water-repellent and self-cleaning surfaces that protect plants in humid environments under high pathogen pressures have recently gained much interest. Those superhydrophobic plant surfaces result from hierarchically aligned structural elements, always including nanoscale wax crystals forming rather fragile and continuously regenerated structures which are often needle-like [1]–[7]. As a consequence, the contact area between the plant surface and liquids or particles is minimized by surface roughness and heterogeneous wetting [8]–[11]. Many recent efforts have tried to mimic key features of superhydrophobic plant surfaces in artificial materials and coatings, but the inherently low mechanical stability of the structures results in rather limited durability [5], [7]. Anti-wetting phenomena are also known from some arthropods and their eggs [12]–[14]. Springtails (Collembola, Entognatha), a wingless arthropod group of more than 7000 species which live in soil, in decaying material, and on plants, have adapted to demanding environmental conditions by evolving extremely effective and robust anti-adhesive skin patterns. They are among the most abundant of all macroscopic animals and considered a separate evolutionary lineage that branched much earlier than the separation of crustaceans and insects [15]. Springtails often live in habitats where water is heavily contaminated by surface-active substances originating from decaying organic matter, and where potentially harmful microorganisms are present [12]. In consequence, they exhibit a very unusual skin structure that reflects an even more pronounced adaptation than that observed in plants [16]–[19]. While it has been previously recognized that the prevention of cuticle wetting is critically important for survival because springtails depend on epidermal respiration previous research efforts have mainly examined the mechanisms Collembola use for surviving drought, freezing and dispersal ability and only few investigations considered the repellent properties of collembolan skin [20]–[24]. Here we explore the structural elements (**Figure 1** **and Figure S1**) of Collembola skin which control interfacial phenomena. We demonstrate that the skin can resist wetting by many organic liquids and at elevated pressures and we suggest a general explanation for the non-wetting characteristics of the related structures.

## Results and Discussion

Scanning electron microscopy studies showed that the skin of springtails exhibits a hierarchical structure of nanoscopic interconnected granules (primary granules) combined with bristles or feathered hairs (**Figure 1**, for *O. stachianus* and *T. bielanensis*, for additional information on 35 different species from 16 families and 4 orders, comprising animals with quite different shape, size and habitat see **Table S1 and Information S1**). 18 out of 35 investigated species were found to possess microscopic, papillous granules (secondary granules). Bristles are tens of microns in length and their hinge-like base allows them to bend in all directions in response to mechanical forces. Distal bristle diameters are very small, ranging from 90 to 150 nm. Comparing the occurrence of secondary granules with the habitat of the related species suggests that these granules mechanically protect the integrity of the nanostructures (for details on the mechanical stability see **Figures S2, S3 and Information S1**). The triangular and quadrangular primary granules of the skin have side lengths of about 200–300 nm and are connected by thinner bars. These connections produce a hexagonal or rhombic comb-like pattern of nanocavities, which covers the whole body of the springtails. The structure size can vary between animals of the same species in response to different environmental influences indicating the capability of the skin to undergo ecomorphological adaptation [25], [26]. In particular, the occurrence of secondary granules was observed on animals living in the soil. Rhombic and hexagonal comb patterns can occur both on the skin of the same animal, whereby the rhombic pattern is present on segments requiring higher elasticity, e.g. for bending.

**Figure 2:**
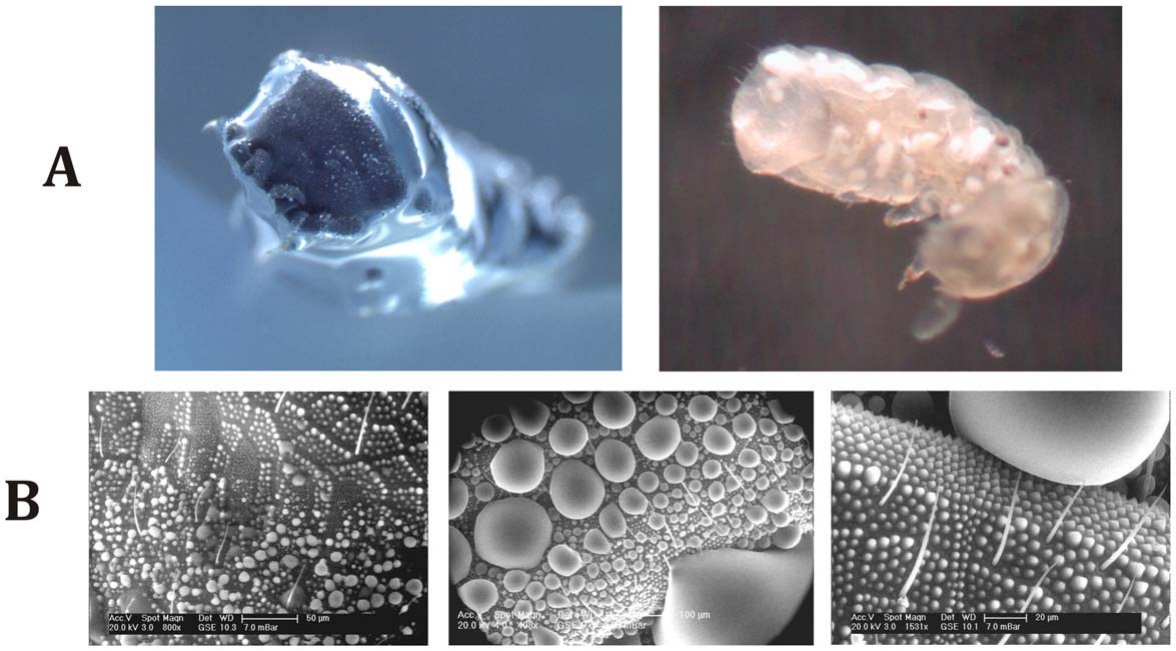
Immersion and water condensation experiments. (A) (left) *T. bielanensis* in water, (right) *Orthonychiurus stachianus* immersed in ethanol resist wetting through the formation of a shiny air cushion. Results of immersion experiments with various liquids (**Table 1**) revealed a resistance of the collembolan skin against wetting by non-polar liquids with surface tensions down to approximately 25 mJ/m^2^. No immersion occurred with any polar liquid. When exposed to increasing pressure, the plastron shrank and the shiny cover disappeared at pressures exceeding 3.5–4.0 bar. After the disappearance of the plastrons, the animals lost their buoyancy and sank. However, different from previously described superhydrophobic surfaces, the shiny plastron reappeared after pressure normalization if the time at reduced pressure did not exceed one minute. This suggests a reversible, pressure-dependent transition between the visible macroplastron and non-visible nanoplastrons enabled by the unique skin topography. (B) At elevated humidity condensation started on the skins of *T. bielanensis*, as a patchy droplet pattern with sizes of around 1 µm (ESEM image). Growing droplets fused or were absorbed by larger drops, leaving behind a completely non-wetted surface on which the described condensation process repeatedly occurred. Repeated droplet fusion finally led to the upward movement of larger drops to the structure tops, which is designated as *anti-fogging*.

To characterize the anti-wetting performance of collembolan skin in some detail, we applied different liquids and condensation experiments. Very stable plastrons (air cushions) were observed around Collembola upon forced immersion in bulk liquids and resist elevated pressures up to values higher than 3.5 atmospheres (**Figures 2** **and Figure S4**). This is in contrast, to the plastron preservation of most other arthropods which was reported to be clearly below two atmospheres [12]. Plastrons of springtails were found to persist for many days and occurred not only in water but also in many polar and non-polar liquids with much lower surface tensions (**Table 1**). Importantly, the collembolan skin not only exhibits superhydrophobicity but similarly superoleophobic characteristics as demonstrated here by the resistance against wetting with a variety of organic liquids, even including tridecane.

**Table 1:** Results of immersion tests of three different species in polar and non-polar liquids.

polar liquids	skin wetting	α [mJ/m^2^]	nonpolar liquids	skin wetting	α [mJ/m^2^]
ethanol	no	22.1	hexane	yes	18.0
methanol	no	22.2	decane	yes	23.5
acetone	no	23.4	cyclohexane	yes	24.7
butanone	no	23.9	dodecane	yes	24.9
1-pentanol	no	25.3	tridecane	no	25.6
2-heptanone	no	26.1	chloroform	no	26.9
Water	no	72.3	hexadecane	no	27.1

Condensation was explored *in situ* under environmental scanning electron microscopy (ESEM) conditions and confirmed the remarkable resistance of collembolan skin against wetting (**Figure 2**). Droplet formation was observed on the secondary granule tops, indicating a stable heterogeneous wetting regime. Even tiny drops with diameters of only a few microns exhibited a spherical shape. Larger droplets occurred in a heterogeneous pattern typically observed with superhydrophobic surfaces and showed contact angles higher than 160° (**Figure 2B**). The fusion of droplets during growth was often accompanied by a lateral displacement of the drops, confirming a very low hysteresis in the wetting behaviour, in line with earlier reports on superhydrophobic surfaces at condensation [27]. The drops formed by this fusion process attained a more spherical shape than the initial drops and a smaller net liquid-solid contact area resulting in durable anti-fogging characteristics of the skin. Together, these features prevent the formation of a continuous water film and thus suffocation of the springtails in high-humidity environments.

**Figure 3:**
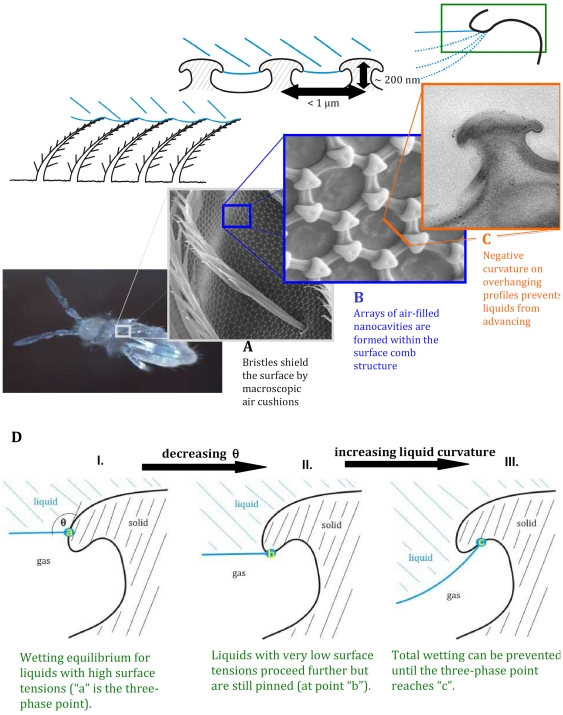
Three levels of protection - the anti-wetting skin morphology of springtails. Multiple design principles are combined to protect collembolan skin against wetting: (A) The hairy cover is the first wetting barrier; liquids can be pinned on the bristle tips. If external forces or very low surface tensions enable liquids to conquer this first barrier, a second principle comes into play: (B) Nanoscopic comb structures of interconnected primary granules can still pin liquids by effective retention of entrapped gas nanobubbles within the surface nanocavities. (C) Gas retention is enforced by the previously unknown fact that the overhanging topographies of the structural elements exhibit a negative curvature (with respect to an orthogonal axis to the surface). The result is a forced Cassie state, through which a dramatically reduced solid–liquid contact area leads to increased macroscopic contact angles of drops on the skin surface. As schematically shown in (D), the design principle protects the surface against wetting independent of the surface chemistry and even at very low surface tensions of the liquid and at elevated pressures.

To further explore the principles behind these unique non-wetting characteristics we analysed the nanoscale features of collembolan skin. Transmission electron microscopy (TEM) revealed overhanging cross-sections with negative curvature of the smallest structural elements; the primary granules and the connecting bars in the mesh structure (**Figure S1**). This peculiar profile enlarges the skin-air interface to facilitate respiration and creates a remarkably strong resistance against wetting according to a previously unknown but surprisingly simple principle: As depicted in **Figure 3**, due to the negative curvature of the overhanging profile, an energy barrier must be overcome by the advancing liquid phase before wetting becomes irreversible even for liquids with very low surface tension. Interestingly, engineered surfaces with isolated microelements containing overhanging profiles were recently reported to exhibit amazing super-oleophobic characteristics and provide -through the variability of structural elements- very valuable insights into design criteria for non-wetting surfaces [28]–[32]. As of now, however, those engineered structures did not include any negative curvatures in the overhanging microelements (as illustrated in **Figure 3**) nor connections of the structural elements, two major components of Collembola skin that explain its uniquely effective and durable anti-adhesive properties. The dimensions of the structural elements of Collembola skin are furthermore at least one magnitude smaller as compared to any of the artificial surfaces considered so far. We suggest that the anti-wetting barrier resulting from the array of nanocavities and the curvature in the shape of the smallest elements was evolutionarily optimized to protect the springtails if acoustic vibrations, pressure jumps or mechanical forces temporarily impose additional energy on the system. While the chemical composition of the springtail skin remains to be analysed in detail, the described design principle can protect surfaces irrespective of their actual chemistry.

Water-repellent, self-cleaning plant surfaces and springtail skin share a hierarchical surface structure with papillous microelements. The collembolan skin, however, is substantially more mechanically stable due to incorporated, flexible bristles and the comb-like alignment of granules (The higher mechanical stability is obvious from the comparison of the surface structures *per se* and confirmed in a sand abrasion experiment described in the supplement, see **Figures S2, S3 and Table S2**.). Due to embedded nanocavities, springtail skin resists wetting more effectively. In line with this, the skin was also found to exhibit outstanding repellence to particles and bacterial or fungal contamination. None of the microscopically investigated samples in our study ever showed a trace of any adhering material. Furthermore, we massively exposed springtails to *Escherichia coli*, *Staphylococcus aureus* and *Candida albicans* (representing Gram-positive bacteria, Gram-negative bacteria and fungi), respectively, for periods of four days under standard culture conditions without observing any significant deposition.

Many valuable opportunities arise from the translation of the described surface structure of collembolan skin into man-made materials and coatings that combine stability against wear and friction with superior non-wetting and anti-adhesive characteristics, addressing critical limitations of the currently employed concepts of superhydrophobic surfaces.

## Materials and Methods

### Animals

*Orthonychiurus stachianus*, *Ceratophysella denticulata* and *Sinella tenebricosa* were collected from the tropical greenhouse at the Dresden Botanical Garden. All necessary permits were obtained for the collection (CN, the director of the Botanical Garden was actively involved in this study) and no endangered or protected species were involved. Petri dishes containing the animals were kept at 21°C and sealed with silicon to prevent escape and dehydration during breeding. Dishes were coated with gypsum mixed with chromite powder (20∶1) to produce a water reservoir and porous substrate that maintained a convenient microclimate. Dark chromite powder was used to support the observation of the white, non-pigmented animals.

*Tetrodontophora bielanensis* was collected from a forest in the mountains of Saxony near Schmilka. No specific permits were required for the collection, the location is not privately-owned nor protected in any way and no endangered or protected species were involved. Large Petri dishes with a silicone seal were used for breeding. For substrate and food, soil, litter, decaying wood and moss from their original habitat were used. Other species – see **Table S1** - were received from the Senckenberg Natural History Museum, Görlitz, Germany.

### SEM/TEM

SEM studies were performed using a XL30 ESEM-FEG microscope (Philips) in the usual HighVac mode at voltages of 10–30 kV. The animals were prepared by exposure to chloroform and subsequent air-drying without fixation. Subsequently they were subsequently coated with a 5–15 nm gold layer (BALZERS SCD 050 Sputter Coater). TEM observations were carried out with a Zeiss EM 912 Omega microscope. Samples were fixed with glutaric aldehyde (C_5_H_8_O_2_) and phosphate buffer [fixative: 10.6 g Na_3_PO_4_+3.5 g K_3_PO_4_+100 ml glutaric aldehyde in 1000 ml H_2_O], and then stained with 2% osmium tetroxide. An increasing acetone series with 1% uranyl acetate was applied for dehydration and staining. After a decreasing acetone/increasing resin series, the samples were embedded in pure resin that was subsequently polymerized in a furnace. Samples were cut with a Leica Ultracut UC6 into ultrathin slices (∼70 nm). Condensation tests were also conducted with the XL30 ESEM-FEG. The condensation process occurred at 1°C under low vacuum (∼10^−1^ mbar) upon slowly increasing the relative humidity until the first droplets were visible.

### Immersion tests

Immersion tests were mainly performed with *Orthonychiurus stachianus*. Polar and non-polar liquids (from Sigma-Aldrich, concentrations ≥99%) were applied to animals in a dish. To slightly force wetting, the liquids were carefully stirred and shaken. In each test, more than 10 animals were used and kept in the liquid for about five minutes.

## Supporting Information

Figure S1 **Characteristic parameters of skin morphology as obtained from SEM and TEM.** Left: TEM image of the skin of *Ceratophysella denticulata*; right: comb structure of *Sinella tenebricosa* (CD …comb diameter, SL … side length of primary granules).
(TIF)

Figure S2 **Sand blast experiment.** Scheme of the abrasion test set-up, SEM image of the applied sand particles.
(TIF)

Figure S3 **Plant and springtail surfaces after sand abrasion tests at varied conditions.** **A** *Colocasia fallax*, **B** *Euphorbia tubifera*, **C** *Limnocharis flava*, **D** *Nelumbo nucifera* and **E** *Xanthosoma violaceum* (left: original; right: **1 cm** height of fall); **F** *Tetrodontophora bielanensis* (left: 3 cm height of fall, same as original; right: **15 cm** dropping height).
(TIF)

Figure S4 **Pressure depending plastron collapse.** Above: scheme of the pressure chamber; below: plot of the stepwise increased pressure with *Orthonychiurus stachianus*.
(TIF)

Table S1 **Springtail skin features of 35 species.**
(PDF)

Table S2 **Sand blast experiment.**
(PDF)

Information S1 **1:** A comparative investigation of 35 species showed a clear ecological and taxonomic dependency of occurrence, size and spacing of the microscaled secondary granules. The alignment of the nanoscaled primary structure elements differed in a more irregular manner over the 16 compared families while the size and spacing of the nanoscopic granules was rather constant. **2:** The analysis of the mechanical stability by a sand abrasion test showed a higher resistance of the springtail skin of *Tetrodontophora bielanensis* compared to five superhydrophobic plant structures. **3:** Tests investigating the plastron collapse under pressure in water confirmed the resistance of springtail skin against forced wetting for three different species. This behavior was found to be independent of the presence of secondary granules on the skin surface.
(DOC)
