# Supplementary figures and images for: Smart Skin Patterns Protect Springtails
Source: PLoS One. 2011 Sep 30;6(9):e25105. doi: 10.1371/journal.pone.0025105 (PMC3184130; doi:10.1371/journal.pone.0025105)

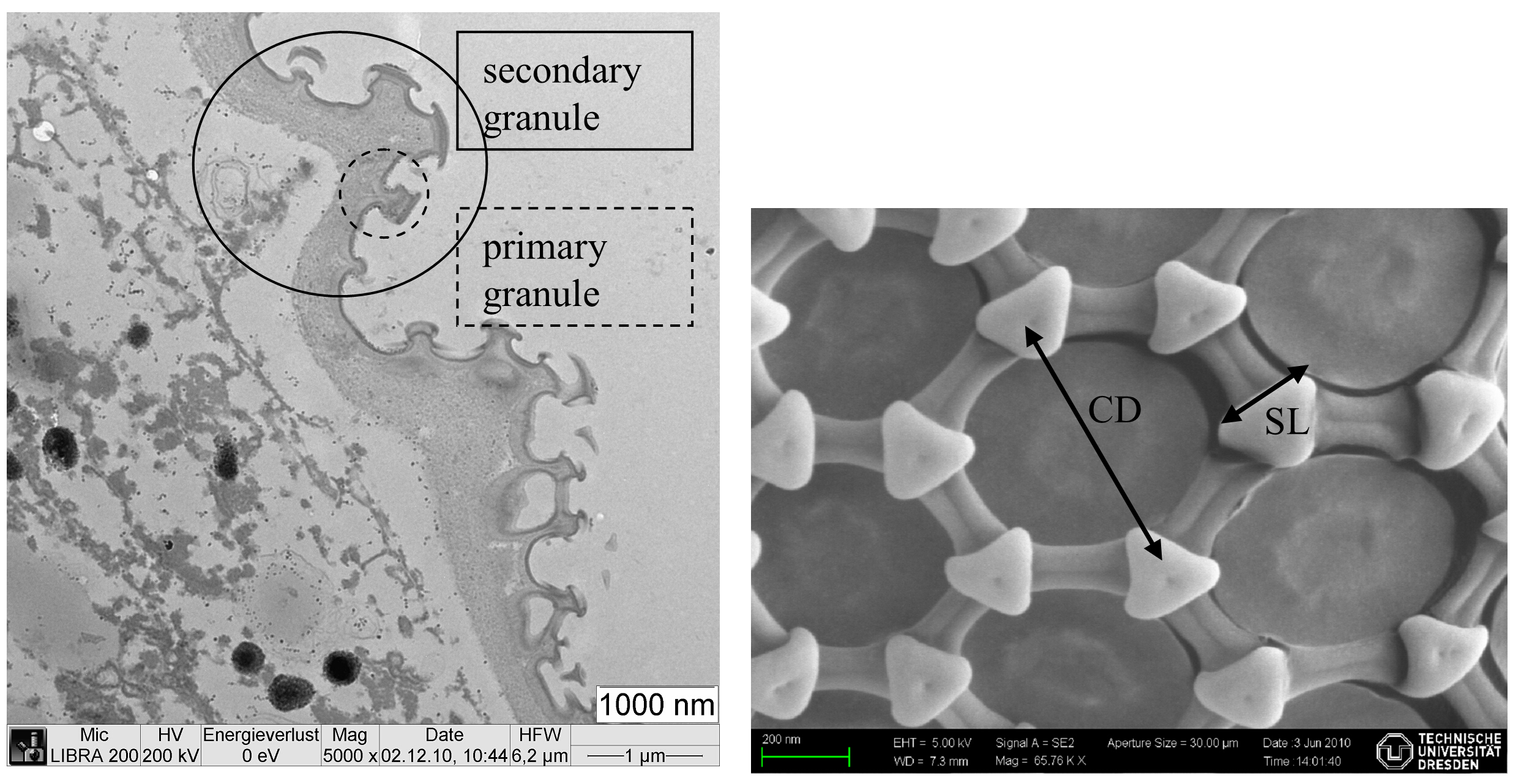

Supplement: Figure S1 — Characteristic parameters of skin morphology as obtained from SEM and TEM. Left: TEM image of the skin of Ceratophysella denticulata; right: comb structure of Sinella tenebricosa (CD …comb diameter, SL … side length of primary granules). (TIF) [file pone.0025105.s001.tif]

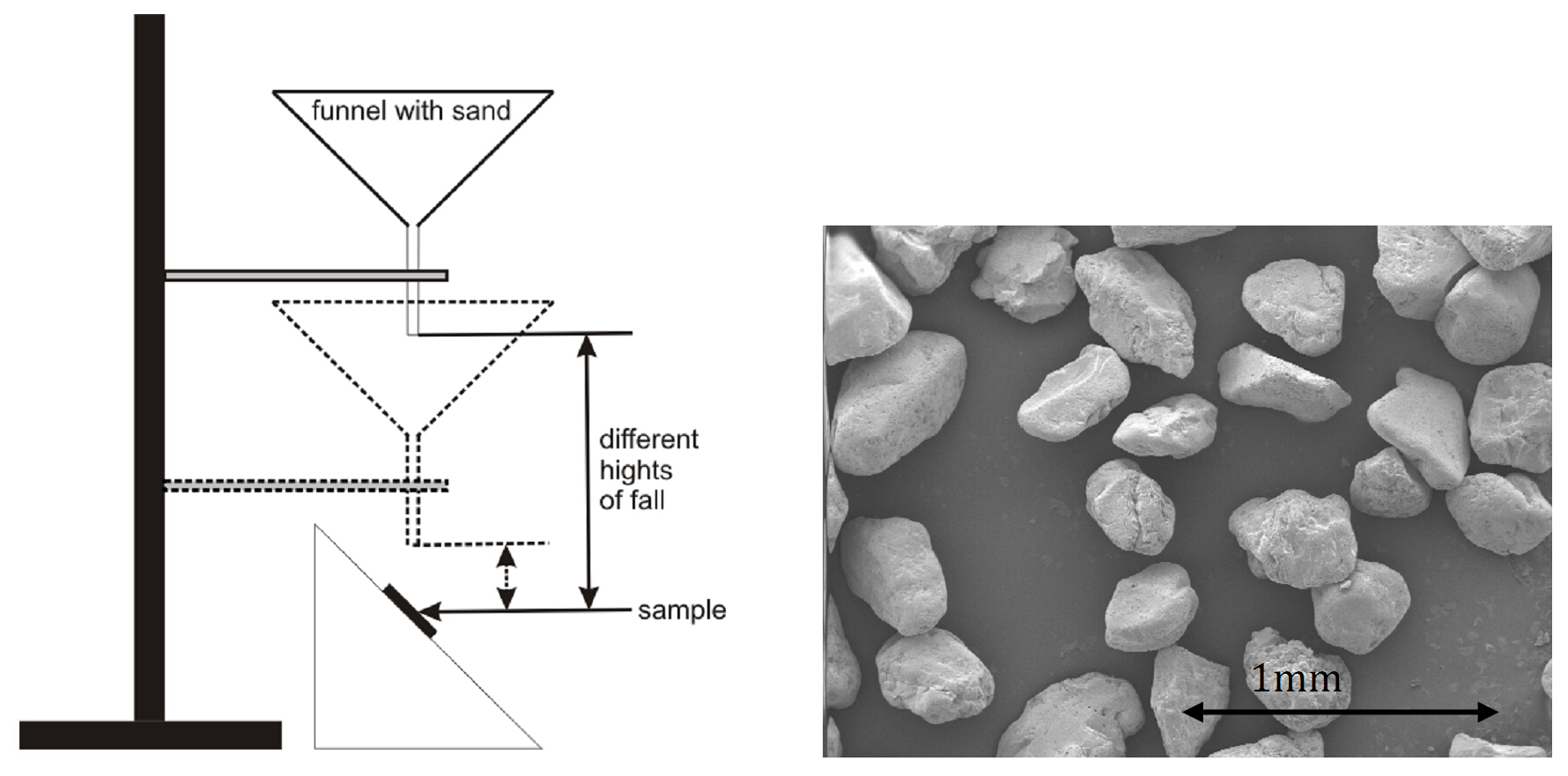

Supplement: Figure S2 — Sand blast experiment. Scheme of the abrasion test set-up, SEM image of the applied sand particles. (TIF) [file pone.0025105.s002.tif]

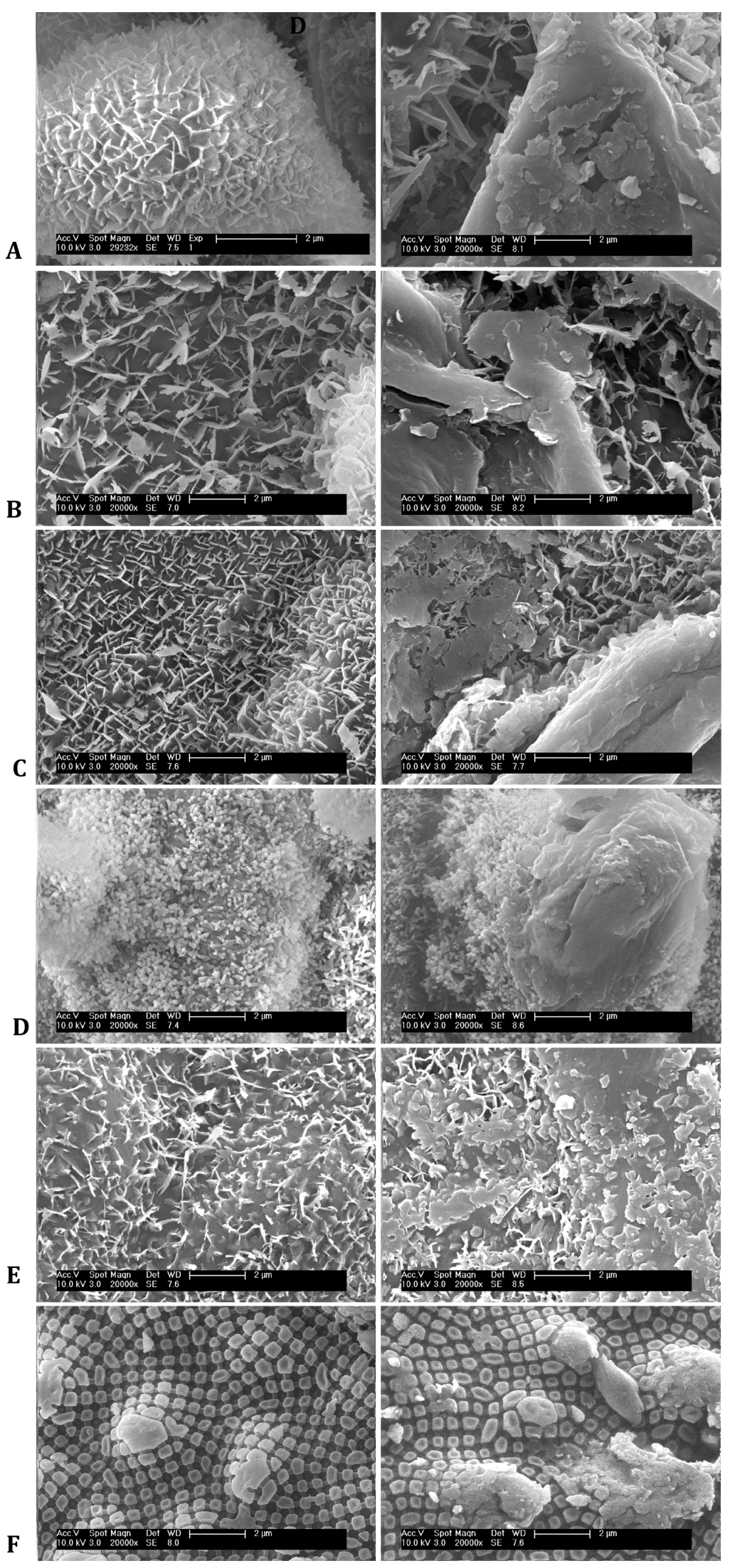

Supplement: Figure S3 — Plant and springtail surfaces after sand abrasion tests at varied conditions. A Colocasia fallax, B Euphorbia tubifera, C Limnocharis flava, D Nelumbo nucifera and E Xanthosoma violaceum (left: original; right: 1 cm height of fall); F Tetrodontophora bielanensis (left: 3 cm height of fall, same as original; right: 15 cm dropping height). (TIF) [file pone.0025105.s003.tif]

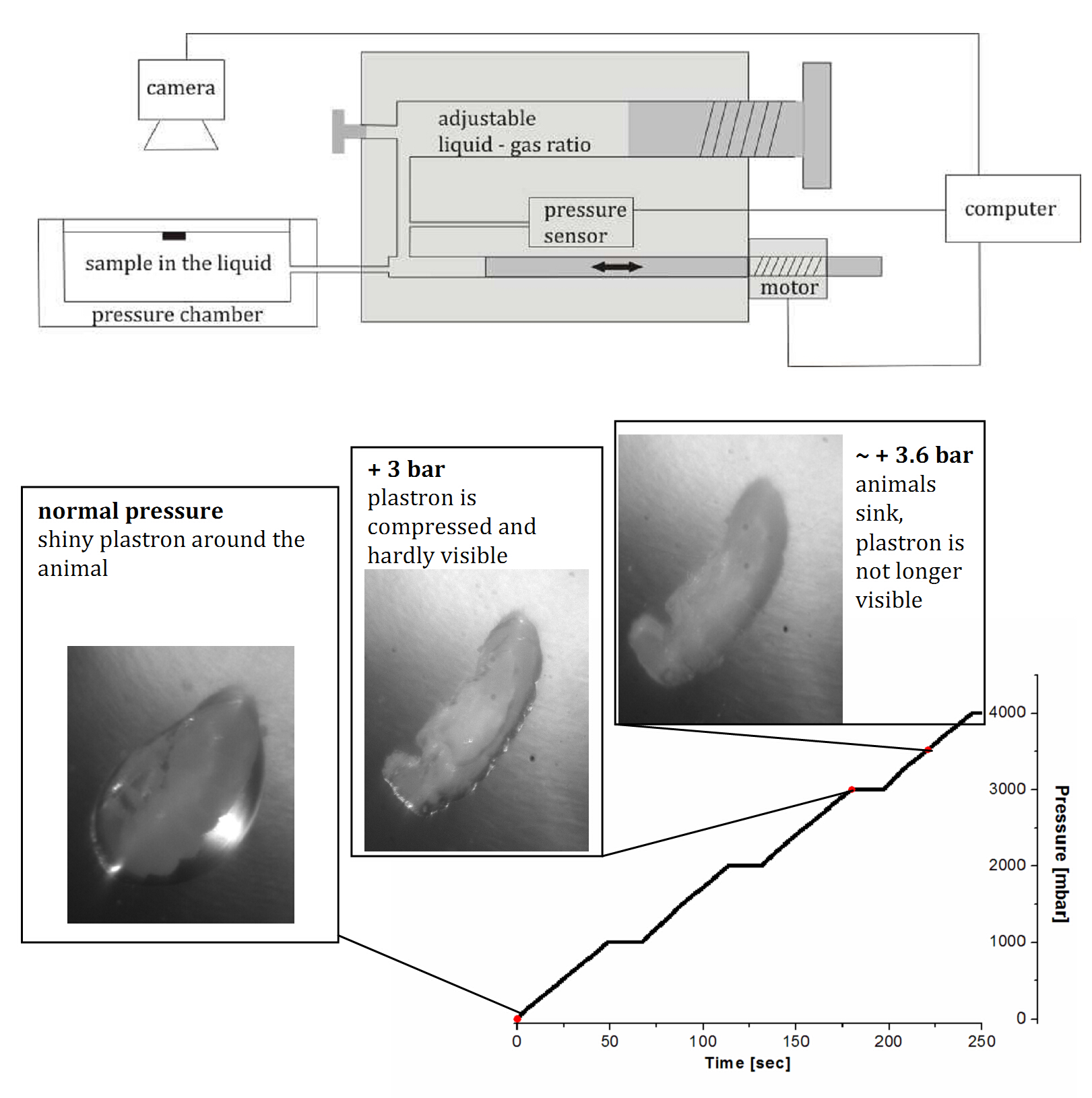

Supplement: Figure S4 — Pressure depending plastron collapse. Above: scheme of the pressure chamber; below: plot of the stepwise increased pressure with Orthonychiurus stachianus. (TIF) [file pone.0025105.s004.tif]
