# Supplementary material for: Smart Skin Patterns Protect Springtails
Source: PLoS One. 2011 Sep 30;6(9):e25105. doi: 10.1371/journal.pone.0025105 (PMC3184130; doi:10.1371/journal.pone.0025105)
Supplement: Table S1 — Springtail skin features of 35 species. (PDF) [file pone.0025105.s005.pdf]

orders (4)	families (16)	species also analyzed by TEM<br>species	main comb alignment	side length of primary granules [nm]	comb diameter [nm]	secondary granules	secondary granule distance [nm]	secondary granule height [nm]
Entomobryomorpha	Entomobryidae	*Entomobrya corticalis* (Nicolet, 1842)	hexagonal	210	1200	no		
	Entomobryidae	*Entomobrya muscorum* (Nicolet, 1842)	hexagonal	180	670	no		
	Entomobryidae	*Sinella tenbricosa* (Folsom, 1902)	hexagonal	200	1200	no		
	Entomobryidae	*Lepidocyrtus paradoxus* (Uzel, 1890)	hexagonal	190	790	no		
	Entomobryidae	*Orchesella flavescens* (Bourlet, 1839)	hexagonal	330	1600	no		
	Entomobryidae	*Seira domestica* (Nicolet, 1842)	hexagonal	190	720	no		
	Tomoceridae	*Pogonognathellus flavescens* (Tullberg, 1871)	hexagonal	220	1000	no		
	Tomoceridae	*Tomocerus minor* (Lubbock, 1862)	hexagonal	180	870	no		
	Oncopoduridae	*Oncopodura crassicornis* (Shoebotham, 1911)	hexagonal	160	620	no		
	Isotomidae	*Hydroisotoma schaefferi* (Krausbauer, 1898)	hexagonal	170	620	no		
	Isotomidae	*Desoria violacea* (Tullberg, 1876)	rhombic	210	570	no		
	Isotomidae	*Folsomia candida* (Willem, 1902)	rhombic	190	440	no		
	Isotomidae	*Folsomia quadrioculata* (Tullberg,1871)	rhombic	180	570	no		
	Isotomidae	*Anurophorus coiffaiti* (Cassagnau & Delamare, 1955)	rhombic	320	700	no		
Poduromorpha	Onychiuridae	*Supraphorura furcifera* (Börner, 1901)	hexagonal & rhombic	200	570	yes	2900	890
	Onychiuridae	*Orthonychiurus stachianus* (Bagnall, 1939)	hexagonal & rhombic	190	550	yes	4700	1800
	Onychiuridae	*Kalaphorura heterodoxa* (Gisin, 1964)	irregular	270	580	yes	7500	5000
	Onychiuridae	*Tetrodontophora bielanensis* (Waga 1842)	rhombic	300	610	yes	5100	3800
	Tullbergiidae	*Stenaphorura quadrispina* (Börner, 1901)	hexagonal	200	590	yes	2800	990
	Tullbergiidae	*Mesaphorura macrochaeta* (Rusek, 1976)	hexagonal	180	600	yes	1400	570
	Tullbergiidae	*Tullbergia callipygos* (Börner, 1902)	hexagonal	240	830	yes	3100	710
	Neanuridae	*Neanura muscorum* (Templeton, 1836)	hexagonal	270	780	yes	6400	4500
	Neanuridae	*Friesa mirabilis* (Tullberg, 1871)	hexagonal	230	650	yes	2200	1500
	Hypogastruridae	*Ceratophysella denticulata* (Bagnall, 1941)	hexagonal	260	810	yes	4300	1200
	Hypogasturidae	*Ceratophysella scotica* (Carpenter & Evans 1899)	hexagonal	280	760	yes	3800	3000
	Brachystomellidae	*Brachystomella parvula* (Schäffer, 1896)	hexagonal	200	620	yes	2200	1800
	Poduridae	*Podura aquatica* (Linneus, 1758)	rhombic	220	470	yes	2200	1400
Symphypleona	Bourletiellidae	*Borletiella hortensis* (Fritch, 1863)	hexagonal & rhombic	350	1200	no		
	Bourletiellidae	*Heterosminthurus insigris* (Reuter, 1876)	hexagonal	220	990	no		
	Sminthuridae	*Allacma fusca* (Linnaeus, 1758)	irregular spikes			no		
	Sminthuridae	*Lipothrix lubbocki* (Tullberg, 1872)	rhombic	290	730	yes	2700	1500
	Dicyrtomidae	*Dicyrtomina ornata* (Nicolet, 1842)	hexagonal & rhombic	260	1000	yes	1500	1200
	Arrhopalitidae	*Arrhopalites pygmaeus* (Wantzel, 1860)	hexagonal & rhombic	190	620	yes	1500	540
	Katiannidae	*Sminthurinus aureus* (Lubbock, 1962)	hexagonal & rhombic	240	870	yes	1600	550
Neelipleona	Neelidae	*Megalothorax minimus* (Willem, 1900)	hexagonal	160	380	yes	950	300
