# Supplementary material for: Smart Skin Patterns Protect Springtails
Source: PLoS One. 2011 Sep 30;6(9):e25105. doi: 10.1371/journal.pone.0025105 (PMC3184130; doi:10.1371/journal.pone.0025105)
Supplement: Table S2 — Sand blast experiment. (PDF) [file pone.0025105.s006.pdf]

**Dropping hight:**	1 cm		3 cm		15 cm	
	visible destructions	loss of antiwetting performance	visible destructions	loss of antiwetting performance	visible destructions	loss of antiwetting performance
**plants**						
Euphorbia tubifera	+	-	+	+	+	+
Limnocharis flava	+	+	+	+	+	+
Nelumbo nucifera	+	+	+	+	+	+
Xanthosoma violaceum	+	+	+	+	+	+
**springtail**						
Tetrodontophora bielanensis	-	-	-	-	+	partial
