# Supplementary material for: Smart Skin Patterns Protect Springtails
Source: PLoS One. 2011 Sep 30;6(9):e25105. doi: 10.1371/journal.pone.0025105 (PMC3184130; doi:10.1371/journal.pone.0025105)
Supplement: Information S1 — 1: A comparative investigation of 35 species showed a clear ecological and taxonomic dependency of occurrence, size and spacing of the microscaled secondary granules. The alignment of the nanoscaled primary structure elements differed in a more irregular manner over the 16 compared families while the size and spacing of the nanoscopic granules was rather constant. 2: The analysis of the mechanical stability by a sand abrasion test showed a higher resistance of the springtail skin of Tetrodontophora bielanensis compared to five superhydrophobic plant structures. 3: Tests investigating the plastron collapse under pressure in water confirmed the resistance of springtail skin against forced wetting for three different species. This behavior was found to be independent of the presence of secondary granules on the skin surface. (DOC) [file pone.0025105.s007.doc]

**Supporting Information S1 for:**

**Smart Skin Patterns Protect Springtails**

Ralf Helbig, Julia Nickerl, Christoph Neinhuis, Carsten Werner

1. Morphological skin features of collembola species from different orders and families

Skin samples from 35 springtail species (four orders, 16 families – **Table S1, Figure S1**) were analyzed by scanning electron microscopy (Zeiss Supra 40 VP - 5 kV high vacuum mode) and from eight species by transmission electron microscopy (Zeiss Libra 200 MC – 200 kV). All preparations were performed as described in the article.

2. Abrasion tests to probe for the mechanical stability of ultrahydrophobic structures

To compare the mechanical stability of the antiadhesive surface nanostructures of springtails and plants a simple abrasion test was performed. 50 g dense rodent bath sand (~2.6 g/cm³) with main grain sizes between 100 and 400 µm was dropped onto samples (~0.25 cm²) which were mounted with double side tape on a tilted ramp (45°) as schematically shown in **Figure S2**. Different dropping heights were adjusted to achieve different impact velocities v [v=(2gx)^1/2^; g and x are the gravitational acceleration and the height of fall respectively]. Leaves from the 5 superhydrophobic plants were received from the Botanical Garden Dresden and cut into pieces (2-4 cm²). *Tetrodontophora bielanensis* were killed before the experiments using ethanol gas.

The surface structures of all plant leaves were found to be massively altered in the abrasion experiment even at the lowest particle drop height (1cm) (**Figure S3**). Four out of five investigated plant species lost their anti-wetting properties already during this treatment (no retention of air cushion upon immersion in water, loss of repellency for water droplets in the treated surface area, see **Table S2**). In contrast, alterations of the springtail skin were only observed when exceeding drop heights of 15 cm.

3. Experiments to analyze the pressure-dependent plastron collapse

The stability of air cushions surrounding springtails at elevated pressures was investigated using a pressure chamber setup as shown in **Figure S4**. The reservoir was compressed and images were recorded using a light microscope during the whole process. To avoid any influence of the gas solubility in water, the experiments were performed at an air/water ratio of 1:1.

Living animals of *Orthonychiurus stachianus*, *Ceratophysella denticulata* and *Sinella tenebricosa* were used (at least three examples per species, from cultures containing adults and juveniles). Two tests in the pressure chamber were performed with all three species. Plastron collapse was observed to occur independent of the investigated species and age. Experiments with *O. stachianus* (shown in **Figure S4**) were repeated four times with different groups with at least 10 certain animals to check for repeatability. The recovery of the plastron was found to strongly depend on the time period between plastron collapse and pressure relaxation. For time periods below 1 minute plastrons recovered for 90% of the animals.

**Table S1 Springtail** skin features of 35 species.

|  | | species also analyzed by TEM | main comb alignment | side length of primary  granules [nm] | comb  diameter [nm] | secondary  granules | secondary granule  distance [nm] | secondary granule  height [nm] |
| --- | --- | --- | --- | --- | --- | --- | --- | --- |
| orders (4) | families (16) | species |
| Entomobryomorpha | Entomobryidae | *Entomobrya corticalis* (Nicolet, 1842) | hexagonal | 210 | 1200 | no |  |  |
|  | Entomobryidae | *Entomobrya muscorum* (Nicolet, 1842) | hexagonal | 180 | 670 | no |  |  |
|  | Entomobryidae | *Sinella tenbricosa* (Folsom, 1902) | hexagonal | 200 | 1200 | no |  |  |
|  | Entomobryidae | *Lepidocyrtus paradoxus* (Uzel, 1890) | hexagonal | 190 | 790 | no |  |  |
|  | Entomobryidae | *Orchesella flavescens* (Bourlet, 1839) | hexagonal | 330 | 1600 | no |  |  |
|  | Entomobryidae | *Seira domestica* (Nicolet, 1842) | hexagonal | 190 | 720 | no |  |  |
|  | Tomoceridae | *Pogonognathellus flavescens* (Tullberg, 1871) | hexagonal | 220 | 1000 | no |  |  |
|  | Tomoceridae | *Tomocerus minor* (Lubbock, 1862) | hexagonal | 180 | 870 | no |  |  |
|  | Oncopoduridae | *Oncopodura crassicornis* (Shoebotham, 1911) | hexagonal | 160 | 620 | no |  |  |
|  | Isotomidae | *Hydroisotoma schaefferi* (Krausbauer, 1898) | hexagonal | 170 | 620 | no |  |  |
|  | Isotomidae | *Desoria violacea* (Tullberg, 1876) | rhombic | 210 | 570 | no |  |  |
|  | Isotomidae | *Folsomia candida* (Willem, 1902) | rhombic | 190 | 440 | no |  |  |
|  | Isotomidae | *Folsomia quadrioculata* (Tullberg,1871) | rhombic | 180 | 570 | no |  |  |
|  | Isotomidae | *Anurophorus coiffaiti* (Cassagnau & Delamare, 1955) | rhombic | 320 | 700 | no |  |  |
| Poduromorpha | Onychiuridae | *Supraphorura furcifera* (Börner, 1901) | hexagonal & rhombic | 200 | 570 | yes | 2900 | 890 |
|  | Onychiuridae | *Orthonychiurus stachianus* (Bagnall, 1939) | hexagonal & rhombic | 190 | 550 | yes | 4700 | 1800 |
|  | Onychiuridae | *Kalaphorura heterodoxa* (Gisin, 1964) | irregular | 270 | 580 | yes | 7500 | 5000 |
|  | Onychiuridae | *Tetrodontophora bielanensis* (Waga 1842) | rhombic | 300 | 610 | yes | 5100 | 3800 |
|  | Tullbergiidae | *Stenaphorura quadrispina* (Börner, 1901) | hexagonal | 200 | 590 | yes | 2800 | 990 |
|  | Tullbergiidae | *Mesaphorura macrochaeta* (Rusek, 1976) | hexagonal | 180 | 600 | yes | 1400 | 570 |
|  | Tullbergiidae | *Tullbergia callipygos* (Börner, 1902) | hexagonal | 240 | 830 | yes | 3100 | 710 |
|  | Neanuridae | *Neanura muscorum* (Templeton, 1836) | hexagonal | 270 | 780 | yes | 6400 | 4500 |
|  | Neanuridae | *Friesa mirabilis* (Tullberg, 1871) | hexagonal | 230 | 650 | yes | 2200 | 1500 |
|  | Hypogastruridae | *Ceratophysella denticulata* (Bagnall, 1941) | hexagonal | 260 | 810 | yes | 4300 | 1200 |
|  | Hypogasturidae | *Ceratophysella scotica* (Carpenter & Evans 1899) | hexagonal | 280 | 760 | yes | 3800 | 3000 |
|  | Brachystomellidae | *Brachystomella parvula* (Schäffer, 1896) | hexagonal | 200 | 620 | yes | 2200 | 1800 |
|  | Poduridae | *Podura aquatica* (Linneus, 1758) | rhombic | 220 | 470 | yes | 2200 | 1400 |
| Symphypleona | Bourletiellidae | *Borletiella hortensis* (Fritch, 1863) | hexagonal & rhombic | 350 | 1200 | no |  |  |
|  | Bourletiellidae | *Heterosminthurus insigris* (Reuter, 1876) | hexagonal | 220 | 990 | no |  |  |
|  | Sminthuridae | *Allacma fusca* (Linnaeus, 1758) | irregular spikes |  |  | no |  |  |
|  | Sminthuridae | *Lipothrix lubbocki* (Tullberg, 1872) | rhombic | 290 | 730 | yes | 2700 | 1500 |
|  | Dicyrtomidae | *Dicyrtomina ornata* (Nicolet, 1842) | hexagonal & rhombic | 260 | 1000 | yes | 1500 | 1200 |
|  | Arrhopalitidae | *Arrhopalites pygmaeus* (Wantzel, 1860) | hexagonal & rhombic | 190 | 620 | yes | 1500 | 540 |
|  | Katiannidae | *Sminthurinus aureus* (Lubbock, 1962) | hexagonal & rhombic | 240 | 870 | yes | 1600 | 550 |
| Neelipleona | Neelidae | *Megalothorax minimus* (Willem, 1900) | hexagonal | 160 | 380 | yes | 950 | 300 |

**Table S2 Sand blast experiment.**

| **Dropping hight:** | 1 cm | | 3 cm | | 15 cm | |
| --- | --- | --- | --- | --- | --- | --- |
|  | visible  destructions | loss of  antiwetting  performance | visible  destructions | loss of  antiwetting  performance | visible  destructions | loss of  antiwetting  performance |
| **plants** |  |  |  |  |  |  |
| *Euphorbia tubifera* | + | - | + | + | + | + |
| *Limnocharis flava* | + | + | + | + | + | + |
| *Nelumbo nucifera* | + | + | + | + | + | + |
| *Xanthosoma violaceum* | + | + | + | + | + | + |
| **springtail** |  |  |  |  |  |  |
| *Tetrodontophora bielanensis* | - | - | - | - | + | partial |
